# An Efficient Quantized Message Passing Receiver Design for SCMA Systems

**DOI:** 10.3390/s25103098

**Published:** 2025-05-14

**Authors:** Hao Cheng, Min Zhang, Ruoyu Su

**Affiliations:** 1 School of Internet of Things, Nanjing University of Posts and Telecommunications, Nanjing 210003, China; haocheng@njupt.edu.cn (H.C.); minzhang@njupt.edu.cn (M.Z.); 2 National Mobile Communications Research Laboratory, Southeast University, Nanjing 210096, China

**Keywords:** bit error ratio, message passing algorithm, quasi-uniform quantization, SCMA

## Abstract

Sparse code multiple access (SCMA) has been considered as an efficient technique to provide both massive connectivity and high spectrum efficiency for future machine-type wireless networks. However, the conventional uniform quantization of the message passing algorithm (MPA) for the SCMA detection induces a significant bit error ratio (BER) performance degradation. In this sense, we propose a new quasi-uniform quantization scheme that can efficiently handle the dynamic range in the exchange of messages. To accelerate the convergence of conventional Max-log MPA, the Sub-log MPA is considered by using the latest updating messages at the current iteration. Simulation results show that the proposed quasi-uniform quantization method can significantly improve the BER performance of the SCMA decoder without modifying the resource nodes’ and variable nodes’ update rules in both the additive white Gaussian noise and the Rayleigh frequency selective channels, as compared to the uniform quantizer.

## 1. Introduction

The upcoming beyond fifth-generation and six-generation wireless networks [1,2] are expected to support hundreds of billions of nodes in the Internet of Things (IoT) [3,4,5], such as the cognitive sensors as well as those found in remote driving [6] and virtual reality, to mention a few. To satisfy the demands of massive connectivity, high throughput and low latency in this IoT scenario, non-orthogonal multiple access (NOMA) [7,8,9,10] has been identified as one of the key physical layer technology to provide higher spectral efficiency and support more connectivities, compared to traditional orthogonal multiple access schemes adopted in the existing 5G systems. The philosophy of NOMA is to allow the simultaneous data transmission for multiple nodes at the same time and frequency resources, and it can be roughly divided into two categories, that is, power domain NOMA (PD-NOMA) and code domain NOMA [11,12,13,14]. In PD-NOMA, distinct transmit power levels are assigned to active users, followed by the superposition coding to multiplex, and the successive interference cancellation technology is applied to eliminate the interference from users with higher power levels in order to detect the transmit symbols of the intended user. Sparse code multiple access (SCMA) [15,16,17,18], multiple user shared access, pattern division multiple access, and interleave division multiple access are several typical schemes of the code domain NOMA [19,20]. In the past decade, significant attention has been directed toward the SCMA, which can be overloaded to enable massive connectivity and achieve high spectrum efficiency. The fundamental principle of SCMA involves mapping the information bits of each user to complex muti-dimensional sparse codewords from its pre-allocated unique codebook and decoding the transmit bits of different users by using the factor-graph-based message passing algorithm (MPA) detector [21].

In communication systems, it has been verified that SCMA can achieve a better sum rate [18] and bit error ratio (BER) [22] performance than PD-NOMA due to the shaping gain of its codewords at the cost of more computational complexity. In this way, various efforts have been made to explore low complexity MPA detectors for SCMA. The original MPA exchanges the message of resource nodes and variable nodes until the maximum number of iterations, which involves redundant calculations. To this end, the authors in [23] present a threshold-based scheme where the reliability of every codeword is computed in each iteration to judge whether the belief threshold is reached. Based on partial marginalization (PM), a fixed low complexity SCMA detector is proposed in [24], in which *t* symbols are fixed in the *m*-th iteration and the computations corresponding to these *t* symbols are not required in the rest iterative calculations. To further reduce its computational complexity, an improved PM-MPA detector [25] is proposed by updating the message more efficiently than the original PM-MPA [24] to obtain reliable codewords. To accelerate its convergence, a shuffled MPA has been proposed in [26] by immediately propagating the updated message between resource nodes and variable nodes in the current iteration. In addition, the authors in [27] also provide a residual-aided message exchanging procedure for scheduling messages in the asynchronous message passing algorithm detector by exploiting the message with the largest residual. The ascending order of the above serial scheduling strategy is not the best selection. In this way, an improved serial scheduling-based MPA method [28] is provided by taking advantage of the most reliable message updates in a timely manner. To address the computational cost challenge in the design of downlink multiple-input multiple-output SCMA systems, the parallel and serial schedules are considered to strike a balance between detector complexity and latency [29]. To enhance the bit error ratio (BER) performance of the SCMA, a scaling factor is introduced to compress likelihood messages [30] to solve the overestimation problem of the iterative multiuser SCMA receiver. By using the lookup table, two variances of the original MPA are provided [31], and these can balance a trade-off between computational complexity and BER performance. The idea of stochastic computing has been introduced into MPA [32] to design novel stochastic logic architectures by carrying out complex computations with very simple logics. In practical, it is essential to integrate SCMA with multi-carrier waveforms to investigate its effectiveness [33,34]. To tackle the peak-to-average power ratio of SCMA combined with orthogonal frequency division multiplexing, a novel clipping noise aided MPA has been taken into consideration for decoding more accurately [33]. By means of the frequency domain implementation of the generalized frequency-division multiplexing (GFDM), a low complexity detection for SCMA-GFDM [34] has been established by applying the discrete Fourier transform operation.

Apart from many solutions for reducing the computational complexity of the conventional MPA, there are several other approaches developed for SCMA detection. By utilizing the lattice structure of its codewords, a low-complexity list sphere decoding has been proposed by avoiding the exhaustive search for all possible hypotheses [35]. Inspired by the Tikhonov regularization, a reduced-complexity optimal modified sphere decoding scheme is provided in [36] by exploiting the sparse structure of the SCMA codebook. The authors in [37] put forward a novel low complexity solution for SCMA detection by adopting an appropriate parameter to narrow down the range of believable superposed constellation points. By sorting the channel matrix and computing a non-zero low bound before searching [38], the proposed modified single tree search-based SCMA detection scheme has lower computational complexity but achieves nearly the same BER performance as compared to the original MPA. However, the above mentioned sphere-decoding-based detectors can only be applied to SCMA with a certain structure of constellations. To address this issue, an improved sphere decoding scheme for SCMA has been provided, and this can serve any arbitrary regular or irregular constellations with the same BER performance as an optimal maximum likelihood detector [39]. Efforts to address the integration of SCMA with other physical techniques, such as multiple-input multiple-output, orthogonal time frequency space, and satellite communications, can be found in [40,41,42,43,44].

Despite many solutions for floating-point SCMA detectors, this paradigm has just emerged for a fixed-point SCMA system [45,46,47], which is more practical for hardware design and implementation. The pioneering work in [45] designs a unified quantization scheme independent of signal-to-noise ratios where the resource nodes’ and variable nodes’ message exchanging are processed synchronously to converge faster. A low complexity attempt has been reported in [46] in the sense that domain changing, probability approximation, early termination, adaptive decoding, and initial noise reduction are employed for SCMA detection, which generates the deterministic message passing algorithm. Moreover, a row-layered message passing algorithm has been provided in [47], which offers a good trade-off between the hardware implementation and BER performance. However, the above structures only consider the uniform quantization strategy, which suffers high complexity or BER degradation in implementations. In particular, the quantization precision is relatively high in order to achieve considerable BER performance; otherwise, the BER suffers a large degradation when using low quantization precision.

To this end, we propose a new quasi-uniform quantizer for the SCMA system instead of the standard uniform one. This quasi-uniform quantizer can efficiently increase the dynamic range of messages and improve the BER performance under the same quantization precision without additional complexity in symbol detection. In other words, the quasi-uniform quantizer can achieve the same BER performance as the uniform one with less quantization bits. The main contributions of this paper are summarized as follows:

- The Sub-log MPA is considered in order to accelerate the convergence of conventional Max-log MPA by splitting the factor graph into two parts and utilizing the latest updating messages at the current iteration.
- Instead of the additive white Gaussian noise (AWGN) channel considered in [45,46,47], we investigate a more general Rayleigh fading channel to describe the standard uniform quantizer.
- To improve the BER performance of SCMA systems, a new quasi-uniform quantizer is formulated without increasing the decoding complexity compared to the traditional one. To the best of our knowledge, this is the first attempt to apply the non-uniform quantizer to the SCMA system.
- Simulation results confirm the validity of our proposed quasi-uniform quantizer, which can significantly reduce the computational complexity to achieve the same BER as the standard one; in other words, it can improve the BER performance as compared to the uniform quantizer by using identical quantization bits.

The rest of the paper is organized as follows. Section 2 introduces the system model for SCMA systems and the conventional log-domain MPA detector. In Section 3, we propose a quasi-uniform quantizer to overcome the limitations of the traditional uniform one. Numerical results are provided in Section 4, followed by conclusions in Section 5.

## 2. System Model

In this section, we first briefly introduce the model of the SCMA system and then describe the framework of the conventional MPA decoder.

### 2.1. SCMA System Description

We consider a typical downlink SCMA system with *K* orthogonal resource nodes (RNs), where *J* randomly distributed users are served by a base station. In general, the SCMA system works in a overloaded manner to achieve better spectral efficiency, and the overloading factor is defined as λ=J/K>1. A unique codebook with cardinality *M* is assigned to each user. At the SCMA modulator, the information bits of the *j*-th user are sequentially mapped into *K*-dimensional complex-valued codewords $x_{j}={\left[x_{1,j},x_{2,j},\cdots ,x_{K,j}\right]}^{T}$. Specifically, this codeword $x_{j}$ is sparse with $d_{v}<K$ nonzero elements, which enables MPA detection for SCMA. In the transmitter, the codeworks $x_{j}$, where j=1,2,⋯,J, from all the *J* users are superimposed and propagate into the air interface. In this way, the received signal $y_{j}={\left[y_{1,j},y_{2,j},\cdots ,y_{K,j}\right]}^{T}$ at the *j*-th user can be described as [48]

(1) $$\begin{array}{c} y_{j}=\mathrm{diag}\left(h_{j}\right)\sum_{i=1}^{J}x_{i}+n_{j}, \end{array}$$

where $h_{j}={\left[h_{1,j},h_{2,j},\cdots ,h_{K,j}\right]}^{T}$ represents the channel gain vector between the base station and the *j*-th user, $\mathrm{diag}(h_{j})$ refers to the diagonal matrix, with the elements in the first diagonal being the components present in $h_{j}$, and $n_{j}={\left[n_{1,j},n_{2,j},\cdots ,n_{K,j}\right]}^{T}$ is the Gaussian noise vector with its elements being modeled as independent and identically distributed $\mathrm{CN}(0,\sigma^{2})$. Thanks to the sparsity of codewords, the sparse structure of SCMA can be represented by a K×J binary indicator matrix F. The element $f_{k,j}$ corresponds to the *k*-th row and *j*-th column of F, and $f_{k,j}=1$ if and only if the *k*-th RN is connected to the *j*-th variable node (VN). In the matrix F, there are $d_{v}$ and $d_{c}$ nonzero elements in each row and column, corresponding to the the number of RNs multiplexed by each VN and the number of VNs accessing each RN. In this sense, we define two sets

(2) $$\begin{array}{c} R_{j}=\left\{k|f_{k,j}=1\right\},j=1,2,\cdots ,J, \end{array}$$

and

(3) $$\begin{array}{c} V_{k}=\left\{j|f_{k,j}=1\right\},k=1,2,\cdots ,K, \end{array}$$

to denote the set of RNs occupied by the *j*-th VN and the set of VNs collided over the *k*-th RN. In this work, two typical indicator matrices for the SCMA system are considered, which support J=6 and J=10 users, respectively, given by [48]

(4) $$\begin{array}{c} F_{4\times 6}=\left[\begin{array}{cccccc} 1 & 1 & 1 & 0 & 0 & 0\\ 1 & 0 & 0 & 1 & 1 & 0\\ 0 & 1 & 0 & 1 & 0 & 1\\ 0 & 0 & 1 & 0 & 1 & 1 \end{array}\right], \end{array}$$

and

(5) $$\begin{array}{c} F_{5\times 10}=\left[\begin{array}{cccccccccc} 1 & 1 & 1 & 1 & 0 & 0 & 0 & 0 & 0 & 0\\ 1 & 0 & 0 & 0 & 1 & 1 & 1 & 0 & 0 & 0\\ 0 & 1 & 0 & 0 & 1 & 0 & 0 & 1 & 1 & 0\\ 0 & 0 & 1 & 0 & 0 & 1 & 0 & 1 & 0 & 1\\ 0 & 0 & 0 & 1 & 0 & 0 & 1 & 0 & 1 & 1 \end{array}\right]. \end{array}$$

**Figure 1:**
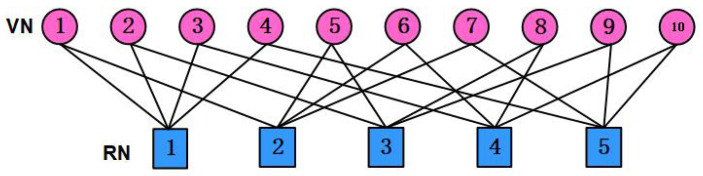
The SCMA factor graph for K=5 and J=10.

The overloading factor of $F_{4\times 6}$ and $F_{5\times 10}$ are λ=150% and λ=200%, respectively. The weights of each column and each row of $F_{4\times 6}$ are $d_{v}=2$ and $d_{c}=3$, while each VN’s degree and each FN’s degree are $d_{v}=2$ and $d_{c}=4$ for $F_{5\times 10}$, respectively. The factor graph to visualize the SCMA configuration in (5) is depicted in Figure 1.

### 2.2. Conventional Log-MPA

The computational load of the conventional MPA probability domain involves a great amount of exponentiation calculation and high storage burden, which limits its implementation in hardware design. To this end, the log domain MPA has been introduced to translate the exponentiation into addition, referred as Log-MPA. This Log-MPA is a bipartite factor-graph-based iterative procedure by exchanging the codeword likelihood messages between variable nodes and resource nodes to achieve a near optimal performance. By defining $R_{j\to k}^{t}$ and $V_{k\to j}^{t}$ as the belief propagated to the *k*-th resource node from the *j*-th variable node and the belief propagated to the *j*-th variable node from the *k*-th resource node at the *t*-th iteration, respectively, the procedure of standard Log-MPA can be described as follows [31].

(1) *Initialization:* Set t=1 and the maximum iteration number *T*. The normalized a priori log-likelihood probability of each *j*-th user’s codeword is assumed to be equal, i.e., $R_{j\to k}^{0}=\log1/M$. Initialize the log-domain conditional probability of different codeword combinations for the *j*-th user as

(6) $$\begin{array}{c} P_{k,j}\left(x\right)=-\frac{1}{2\sigma^{2}}{\left|y_{k,j}-h_{k,j}\sum_{m\in V_{k}}x_{k,m}\right|}^{2}, \end{array}$$

where $x=\left\{x_{m}\right\},m\in V_{k}$.

(2) *Resource Node Updating*: The belief propagated to the *j*-th variable node from the *k*-th resource node at the *t*-th iteration can be calculated as

(7) $$\begin{array}{c} V_{k\to j}^{t}\left(x_{j}\right)=\max_{x_{u}:u\in V_{k}\setminus j}^{\;\;\;\;\;\;*}\left\{P_{k,j}\left(x\right)+\sum_{u\in V_{k}\setminus j}R_{u\to k}^{t-1}\left(x_{u}\right)\right\}, \end{array}$$

where $V_{k}\setminus j$ represents the $d_{c}-1$ VNs associating with the *k*-th RN with the *j*-th VN excluded, and

(8) $$\begin{array}{c} \max^{\;\;\;\;\;\;*}\left(a_{1},a_{2},\cdots ,a_{n}\right)=\ln\left(e^{a_{1}}+e^{a_{2}}+\cdots +e^{a_{n}}\right). \end{array}$$

(3) *Variable Node Updating*: Resource nodes transmit the updated information obtained from extrinsic information to their neighboring variable nodes, given by

(9) $$\begin{array}{c} R_{j\to k}^{t}\left(x_{j}\right)=\sum_{m\in R_{j}\setminus k}V_{m\to j}^{t-1}\left(x_{j}\right), \end{array}$$

where $R_{j}\setminus k$ represents the $d_{v}-1$ RNs associating with the *k*-th VN with the *j*-th RN excluded.

(4) *Probability Calculating and Symbol Judging*: When the maximum number of iterations is reached, the final probabilities of each codeword for each user are output. The final probabilities of codeword $x_{j}$ for VN *j* are the sum of all the messages from its neighboring RNs, which can be computed as

(10) $$\begin{array}{c} R_{j}\left(x_{j}\right)=\sum_{m\in R_{j}}V_{m\to j}^{T}\left(x_{j}\right). \end{array}$$

The final estimated transmit codeword $\hat{x_{j}}$ of user *j* is the one which maximizes $R_{j}\left(x_{j}\right)$.

## 3. Proposed Quasi-Uniform Quantizer for SCMA System

In this section, we first present the Max-log MPA detector by utilizing the Jacobi formula. The sub-factor-graph-based Max-log MPA scheme (Sub-log MPA) is then introduced by splitting the factor graph into two parts to accelerate its convergence. Next, we describe the standard uniform quantizer for the SCMA system. Finally, the quasi-uniform quantizer is proposed to enhance the range and precision of the quantized messages during iteration.

### 3.1. Max-Log MPA Detector for the SCMA System

Observe (7), it is obvious that the computational complexity of Log-MPA mainly lies in the exponential operation within $\max^{*}\left(\cdot \right)$ in the VN message updating. Note that

(11) $$\begin{array}{c} \max^{\;\;\;\;\;\;\;*}\left\{a_{1},a_{2}\right\}=\ln\left(e^{a_{1}}+e^{a_{2}}\right)=\max\left\{a_{1},a_{2}\right\}+\ln\left(1+e^{-|a_{1}-a_{2}|}\right), \end{array}$$

where max{·} is the maximum operation, $\ln(1+e^{|a_{1}-a_{2}|})$ is the correction element, and this element can be eliminated. For the case with more than two numbers, by recursively applying this equation, the Jacobian approximation [49,50] can be obtained as

(12) $$\begin{array}{c} \max^{\;\;\;\;\;\;\;*}\left\{a_{1},a_{2},\cdots ,a_{n}\right\}\approx \max\left\{a_{1},a_{2},\cdots ,a_{n}\right\}. \end{array}$$

By using the Jacobian approximation above, the resource node updating in (7) can be simplified as [31]

(13) $$\begin{array}{c} V_{k\to j}^{t}\left(x_{j}\right)\approx \max_{x_{u}:u\in V_{k}\setminus j}\left\{P_{k,j}\left(x\right)+\sum_{u\in V_{k}\setminus j}R_{u\to k}^{t-1}\left(x_{u}\right)\right\}. \end{array}$$

In this sense, we achieve the low complexity implementation of Max-log MPA for SCMA detection.

### 3.2. Sub-Log MPA Detector for the SCMA System

**Figure 2:**
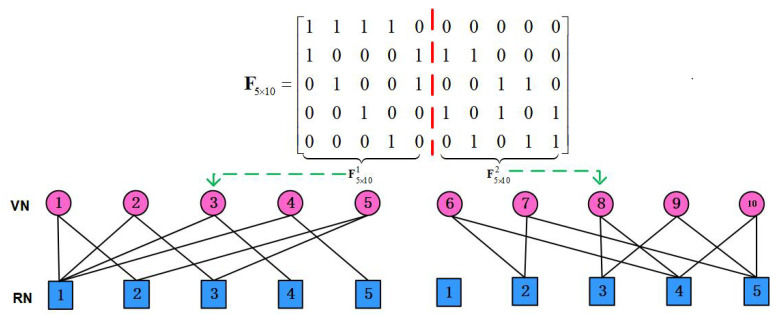
The SCMA sub factor graph for K=5 and J=10.

As discussed above, Max-log MPA can avoid exponent calculation compared to the conventional probability domain MPA, which significantly reduces the computational cost and makes the hardware implementation feasible. However, the number of iterations required to achieve convergence in Max-log MPA is relatively large, which limits the hardware performance and makes the decoding latency high. Moreover, based on (9) and (13), it is obvious that both the RN and VN message exchanging at the *i*-th iteration use the probabilities obtained from the i−1-th iteration. However, the latest probabilities from the current iteration’s updating of the VNs can be immediately utilized to take part in the message propagation of the remaining RNs. In this way, it utilizes more reliable probabilities in the message iteration than the Max-Log algorithm at the same iteration, which can efficiently accelerate the convergence behavior. To achieve this, the indicator matrix can be divided into two parts, where the first part only uses the probabilities obtained from the i−1-th iteration, while the second part can immediately use the more reliable probabilities in the current iteration from the first part. This operation reduces the switch between the RN and VN message propagation, and subsequently decreases the difficulty of hardware design. As depicted in Figure 2, the indicator matrix $F_{5\times 10}$ can be divided into $F_{5\times 10}^{1}$ and $F_{5\times 10}^{2}$, and the sub factor graph are also illustrated. In this way, the message exchanging between RNs and VNs at the *i*-th iteration can be split into two steps, corresponding to the $F_{5\times 10}^{1}$ and $F_{5\times 10}^{2}$, respectively. The first step to conduct RN and VN updating at the *i*-th iteration can be formulated as

(14) $$\begin{array}{c} V_{k\to j}^{t}\left(x_{j}\right)=\max_{x_{u}:u\in V_{k}\setminus j,j\in \left[1,J/2\right]}\left\{P_{k,j}\left(x\right)+\sum_{u\in V_{k}\setminus j}R_{u\to k}^{t-1}\left(x_{u}\right)\right\},j\in \left[1,J/2\right], \end{array}$$

and

(15) $$\begin{array}{c} R_{j\to k}^{t}\left(x_{j}\right)=\sum_{m\in R_{j}\setminus k}V_{m\to j}^{t-1}\left(x_{j}\right),j\in \left[1,J/2\right]. \end{array}$$

The probabilities corresponding to the remaining variable nodes of the second factor graph are then updated as

(16) $$\begin{array}{c} V_{k\to j}^{t}\left(x_{j}\right)=\max_{x_{u}:u\in V_{k}\setminus j,j\in \left[J/2+1,J\right]}\left\{P_{k,j}\left(x\right)+\sum_{u\in V_{k}\setminus j}R_{u\to k}^{t-1}\left(x_{u}\right)\right\},j\in \left[J/2+1,J\right], \end{array}$$

and

(17) $$\begin{array}{c} R_{j\to k}^{t}\left(x_{j}\right)=\sum_{m\in R_{j}\setminus k}V_{m\to j}^{t-1}\left(x_{j}\right),j\in \left[J/2+1,J\right]. \end{array}$$

The implementation of the proposed Sub-log MPA detector for the SCMA system is summarized in Algorithm 1. Note that, different from the parallel and serial schedules adopted in [29], where all the nodes pass new messages to their neighbors, this Sub-log MPA detector only exchanges the new messages to the second sub factor graph, which is more effective in hardware design by exchanging the probabilities among the two sub factor graphs.
**Algorithm 1:** The iterative implementation of Sub-log MPA**Input**: $y_{j},h_{j},\sigma^{2},T$  1**Initialization**  2Initialize the log-domain conditional probability of different codeword  combinations for the *j*-th user as (6).  3**Iteration**  4**for** t=1:T **do**  5      Conduct RN and VN updating at the *i*-th iteration for the first sub factor graph        according to (14) and (15)  6      Update the probabilities corresponding to the remaining variable nodes        according to (16) and (17).  7**end**(  8**Probability Calculation**  9**for** j=1:J **do**(10      When the maximum number of iterations *T* is reached, the final probabilities of        each codeword for each user are computed according to (10).11**end**(

### 3.3. Complexity Analysis

From the above discussion, it is obvious that the difference between the original Log-MPA and the Max-log MPA lies on the resource node updating, i.e., Equations (8) and (12). It is straightforward that the message exchange in (8) consists of a large number of exponential and logarithmic operations, while the RN updating in (12) only contains a comparison operation, which significantly reduces the computational cost of the original SCMA detector. On the other hand, the computational complexity of the Max-log MPA is identical to that of the Sub-log MPA in each iteration. However, the Sub-log MPA in Section 3.2 needs a lower number of iterations to converge due to its utilization of the latest updating messages in the current iteration, which subsequently reduces the computational cost of the Max-log MPA. Based on (14) and (16), it is clear that the storage overhead of the Sub-log MPA is identical to that of the Max-log MPA.

### 3.4. The Standard Uniform Quantizer for SCMA

By defining the quantization step Δ, for a real number *a*, the quantizer can be described as

(18) $$\begin{array}{c} Q\left(a,\Delta \right)=sign\left(a\right)\Delta \left\lfloor \frac{\left|a\right|}{\Delta }+0.5\right\rfloor , \end{array}$$

where sign(a) determines the sign of a real number *a* and ⌊a⌋ outputs an integer not larger than *a*. It is obvious that the output of Q(a,Δ) in (18) obeys the form of lΔ, where *l* is an integer. For a *q*-bit standard uniform quantizer, the quantization results can be expressed as

(19) $$\begin{array}{c} Q\left(a,\Delta \right)=\left\{\begin{array}{c} L\Delta ,\;\;\;\;\;\;\;\;\;\;\;a\geq (L-0.5)\Delta \\ l\Delta ,\;\;\;(l-0.5)\Delta \leq a<(l+0.5)\Delta ,0<l<L\\ 0,\;\;\;\;\;\;\;\;\;\;-\Delta /2<a<\Delta /2\\ l\Delta ,\;\;\;(l-0.5)\Delta <a\leq (l+0.5)\Delta ,-L<l<0\\ -L\Delta ,\;\;\;\;\;\;\;\;\;a\leq -L\Delta +\Delta /2 \end{array}\right., \end{array}$$

where $L=2^{q-1}-1$ and its output alphabet contains $2L+1=2^{q}-1$ number of elements, given by

(20) $$\begin{array}{c} \{-L\Delta ,(-L+1)\Delta ,\cdots ,-\Delta ,0,\Delta ,\cdots ,(L-1)\Delta ,L\Delta \}. \end{array}$$

Note that the *q* binary bits are composed of (*q* − 1) bits to apply binary representation and one sign bit with a value of 0 when *l* is positive, otherwise with a value of 1.

### 3.5. The Proposed Quasi-Uniform Quantizer for SCMA

In the aforementioned standard uniform quantizer, there are two approaches to enhance the performance of the SCMA detector. One is to increase the quantization precision without changing the quantization step Δ, i.e., increase the value of *q*, which put forward higher requirements for the hardware implementation. Another method is to increase the quantization step Δ, which can expand the range of quantized probabilities, which scarifies the precision in application. To solve these problems, we aim to design a quasi-uniform quantizer that can handle the conditions below.

- A large dynamic range of the probabilities is allowed.
- The quantization precision is less than the standard uniform quantizer; i.e., the quasi-uniform quantizer can achieve the same BER performance as the standard uniform quantizer with less quantized bits.
- The hardware implementation complexity can be controlled; in fact, the proposed quasi-uniform quantizer has the same hardware implementation complexity as the standard uniform one. The difference between the quasi-uniform and uniform quantizers lies in the quantization thresholds, i.e., the quantization thresholds of the uniform quantizer is uniformly distributed, while it becomes non-uniformly distributed for the quasi-uniform quantizer.

To this end, we propose a quasi-uniform quantizer for SCMA detection, where the uniform quantization role is adopted at the low region of the probabilities and the nonuniform quantizer is applied at the high region of the messages. To describe this quantizer, we call it the q+1-bit quasi-uniform quantizer. Specifically, for the input values −μLΔ<a<μLΔ, the uniform quantizer in (19) is used, while for the values in the intervals (−∞,μLΔ] and [μLΔ,+∞), the outputs of this quasi-uniform quantizer are in the form of $\pm \mu^{m}L\Delta$, i.e., the corresponding intervals increase exponentially in length with growth rate μ. Note that 1≤m≤L+1; that is to say, there are $2\left(L+1\right)=2^{q}$ values in the form of $\mu^{m}L\Delta$, which can expand the range of the quantized probabilities efficiently. In this way, the q+1-bit quasi-uniform quantizer can be defined as follows.

(21) $$\begin{array}{c} Q_{n}\left(a,\Delta \right)=\left\{\begin{array}{c} \mu^{L+1}L\Delta ,\;\;\;\;\;a\geq \mu^{L+1}L\Delta ,\\ \mu^{m}L\Delta ,\;\;\;\;\mu^{m}L\Delta \leq a<\mu^{m+1}L\Delta ,1\leq m\leq L\\ L\Delta ,\;\;\;\;(L-0.5)\Delta \leq a<\mu L\Delta \\ l\Delta ,\;\;\;(l-0.5)\Delta \leq a<(l+0.5)\Delta ,0<l<L\\ 0,\;\;\;\;\;\;\;\;\;\;\;-\Delta /2<a<\Delta /2\\ l\Delta ,\;\;\;(l-0.5)\Delta <a\leq (l+0.5)\Delta ,-L<l<0\\ -L\Delta ,\;\;\;\;\;\;\;\;\;\;\;a\leq -L\Delta +\Delta /2\\ -\mu^{m}L\Delta ,\;\;\;\;-\mu^{m+1}L\Delta <a\leq -\mu^{m}L\Delta ,1\leq m\leq L\\ -\mu^{L+1}L\Delta ,\;\;\;\;\;a\leq -\mu^{L+1}L\Delta \end{array}\right. \end{array}$$

Note that the q+1 binary bits are composed of *q* bits to apply binary representation and one sign bit. For a comprehensive treatment of the qusi-uniform quantizer, the example of (4+1)-bit quasi-uniform quantization with Δ=0.5,q=3,μ=2 is shown in Table 1. To simplify the representation, only non-negative inputs are considered in Table 1, where the first bit is the sign bit. Based on Table 1, the proposed (4+1)-bit quasi-uniform quantizer operates the same as the 4-bit uniform quantizer in the interval [0,8), while it works in the exponentially increasing way when a≥8.

## 4. Simulation Results

In this section, we provide the BER performance of different SCMA detectors and different quantized bits under various signal-to-noise ratios (SNRs). Both the additive white Gaussian noise (AWGN) and Rayleigh channels are investigated. In our simulations, two factor graphs are considered, and the corresponding index matrices, $F_{4\times 6}$ and $F_{5\times 10}$, are given in (4) and (5), respectively. The channel gain $h_{j}$ was modeled as an independently and identically distributed circularly symmetric complex-valued Gaussian random vector with zero mean and covariance $\sigma^{2}I$.

### 4.1. BER Evolution for AWGN Channel

**Figure 3:**
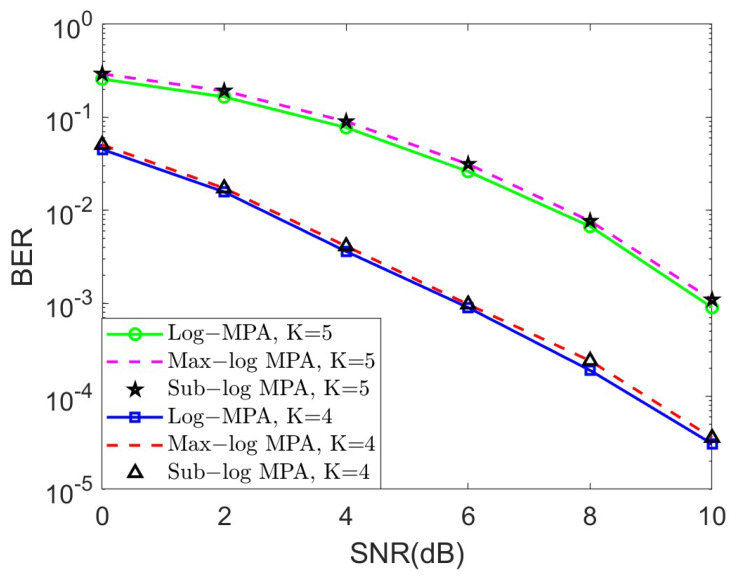
The BER performance of different SCMA detectors under the AWGN channel for K=4 and K=5, respectively.

In this subsection, we examine the validity of our proposed quasi-uniform quantizer for the SCMA system over the AWGN channel. In Figure 3, the bit error ratio (BER) performance of different SCMA detectors under the AWGN channel was observed for K=4 and K=5, respectively. The steps of the Sub-log MPA detector have been summarized in Algorithm 1, and the iteration number is set to 10. It is obvious that the BER performance gap between the original Log-MPA and the Max-log MPA can be neglected, but the Max-log MPA can achieve less computational complexity, which is essential to practical implementation. Note that the curve of the Sub-log MPA is overlapped with the Max-log MPA in bot cases, which in return verifies the effectiveness of the proposed Sub-log MPA.

**Figure 4:**
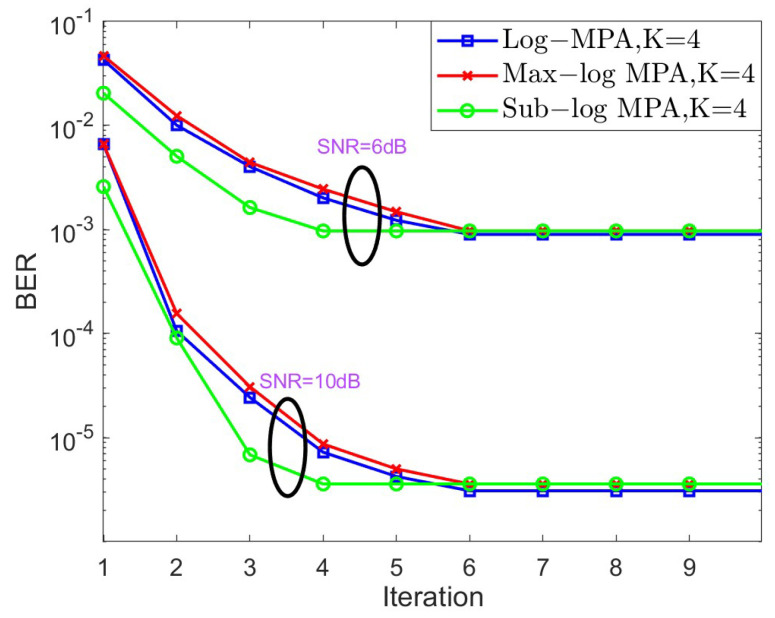
The convergence behavior of different SCMA detectors under the AWGN channel for K=4.

**Figure 5:**
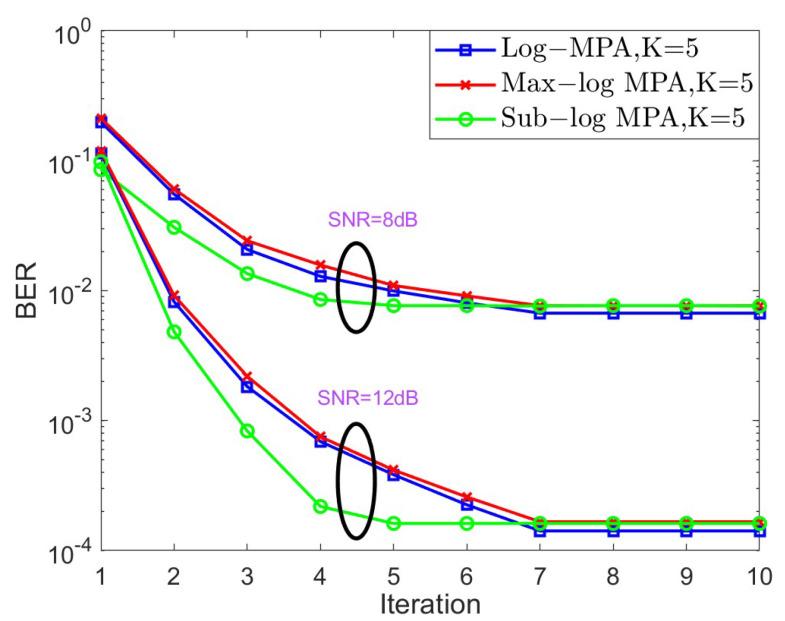
The convergence behavior of different SCMA detectors under the AWGN channel for K=5.

In the next stage, the convergence behavior of different SCMA detectors is shown in Figure 4 and Figure 5 for K=4,J=6 and K=5,J=10, respectively, over the AWGN channel in the context of different SNRs. For the case of K=4, SNR = 6 dB and SNR = 10 dB are taken into account, while SNR = 8 dB and SNR = 12 dB are considered. Based on Figure 4 and Figure 5, it is obvious that both the original Log-MPA and the Max-log MPA need six iterations to converge, while only four iterations are enough for the Sub-log MPA. This is because the Sub-log MPA uses the latest updating message in the current iteration to exchange probabilities.

**Figure 6:**
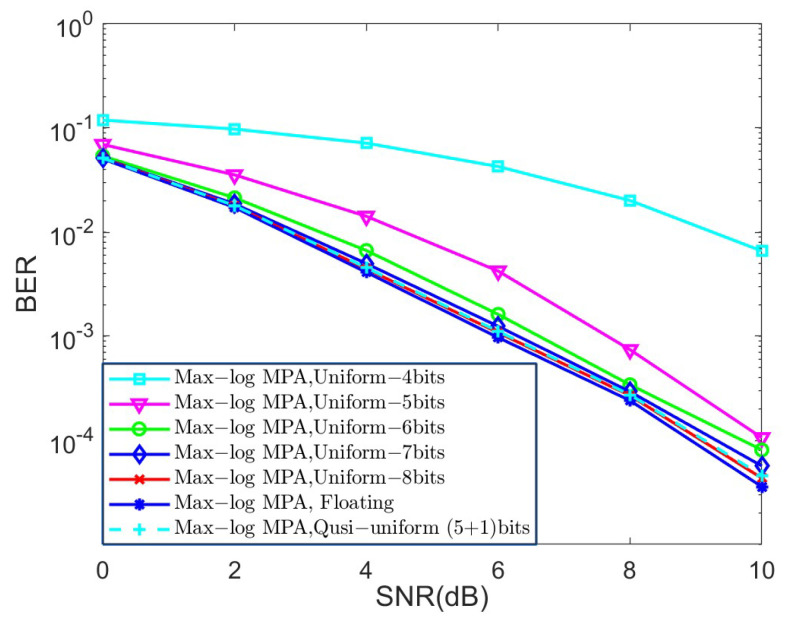
The BER performance of different quantized schemes under the AWGN channel for K=4 and the Max-log MPA detector.

**Figure 7:**
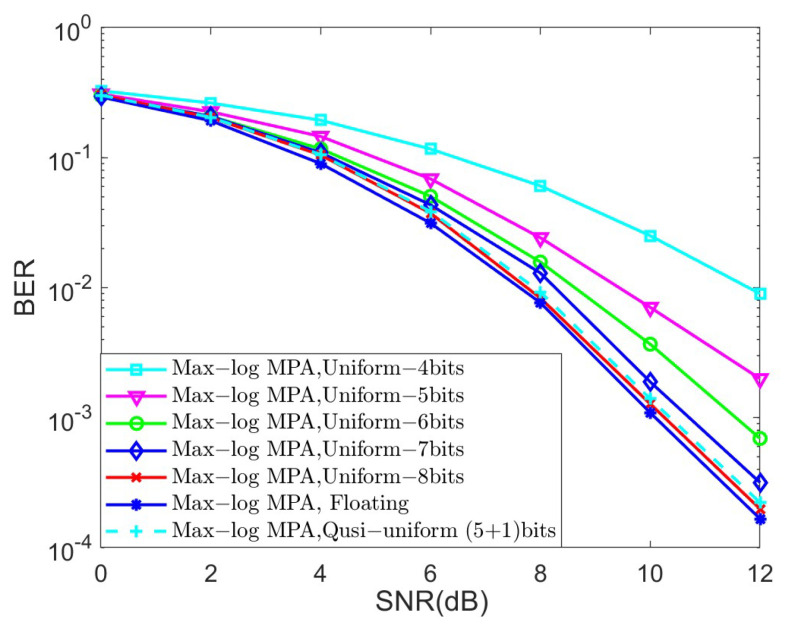
The BER performance of different quantized schemes under the AWGN channel for K=5 and the Max-log MPA detector.

**Figure 8:**
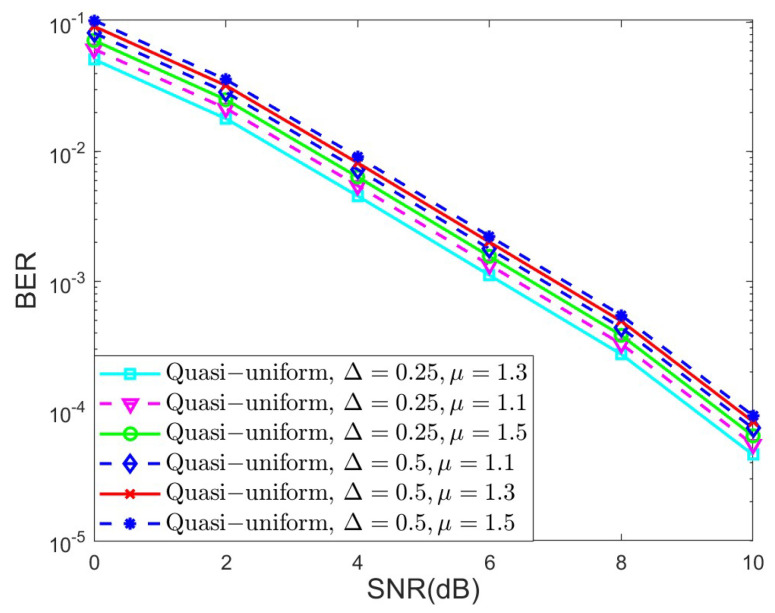
The BER performance of the (5+1)-bit quasi-uniform quantizer under the AWGN channel for K=4 and the Max-log MPA detector, under different combinations of Δ and μ.

In the last set of simulations, we investigated the BER performance of different quantized schemes under the AWGN channel for K=4 and K=5, as shown in Figure 6 and Figure 7, respectively, in terms of the Max-log MPA detector. The parameters of the quasi-uniform quantizer were set to Δ=0.25 and μ=1.3. The uniform and quasi-uniform quantizer were obtained according to Equations (19) and (21). Based on Figure 6 and Figure 7, it is obvious that the BER performance suffers great degradation when the 4-bit, 5-bit, and 6-bit quantizers were considered. This is because the range of quantized messages is limited with the fixed step size Δ. Notably, the 8-bit uniform quantization of the messages can achieve almost the same BER performance as compared to the floating Max-log MPA, while the (5 + 1)-bit quasi-uniform quantization have nearly the same BER performance as that of the 8-bit uniform quantization. Moreover, the BER performance of the (5 + 1)-bit quasi-uniform quantizer under the AWGN channel for K=4 and the Max-log MPA detector, under different combinations of Δ and μ, was investigated in Figure 8. Figure 8 shows that it achieves better BER performance when Δ=0.25 and μ=1.3, as compared to other cases. In general, the values of Δ and μ should be selected according to the simulation results when the number of *J* and *K* becomes larger. All the simulations above verify the robustness of our proposed quasi-uniform quantizer.

### 4.2. Ber Evolution for the Rayleigh Channel

**Figure 9:**
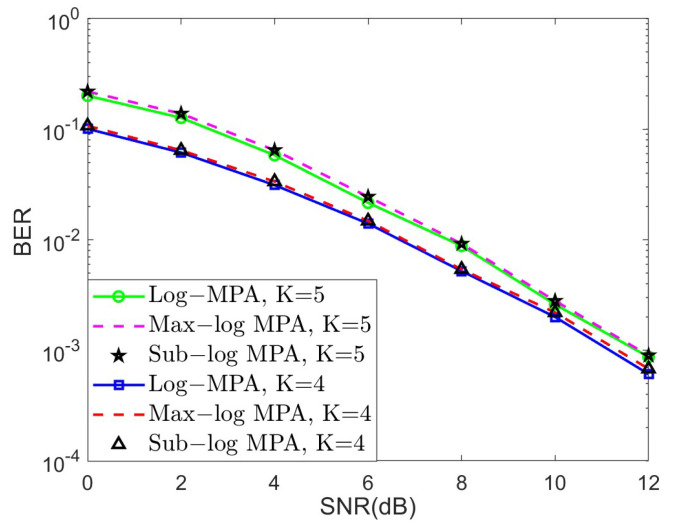
The BER performance of different SCMA detectors under the Rayleigh channel for K=4 and K=5, respectively.

**Figure 10:**
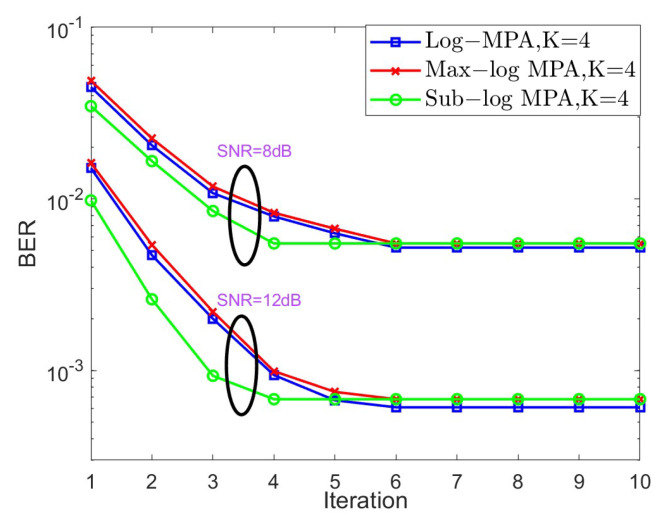
The convergence behavior of different SCMA detectors under the Rayleigh channel for K=4.

**Figure 11:**
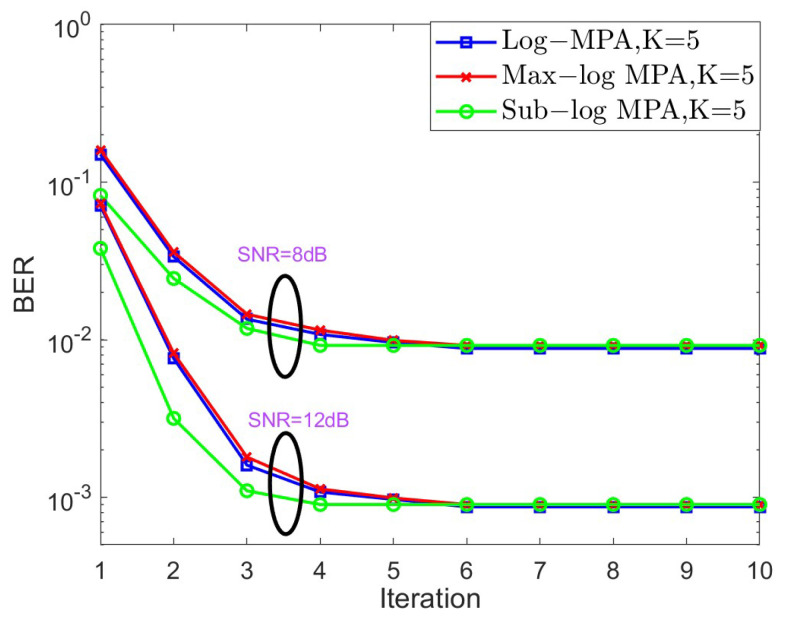
The convergence behavior of different SCMA detectors under the Rayleigh channel for K=5.

In this subsection, numerical results were conducted for different SCMA detectors for the Rayleigh channel. Figure 9 illustrated the BER performance of different SCMA detectors under the Rayleigh channel for K=4 and K=5, respectively. The effectiveness of the Max-log MPA and Sub-log MPA can be observed. Specifically, the BER curves of Max-log MPA and Sub-log MPA are overlapped, and their robust performance can be inferred from a close match to the original Log-MPA detector. Next, we investigated the convergence behavior of different SCMA detectors for the Rayleigh channel in Figure 10 and Figure 11 in terms of K=4 and K=5, respectively. It can observed that the Sub-log MPA only needs four iterations to converge, while six are needed both in the Log-MPA and Max-log MPA schemes.

**Figure 12:**
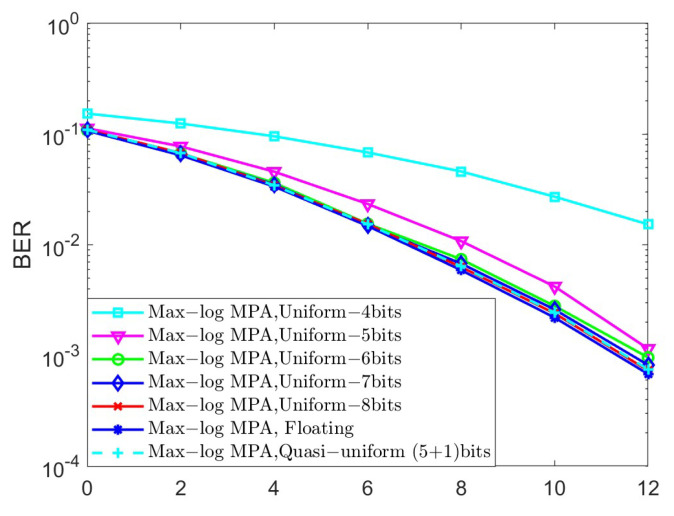
The BER performance of different quantized schemes under the Rayleigh channel for K=4 and the Max-log MPA detector.

**Figure 13:**
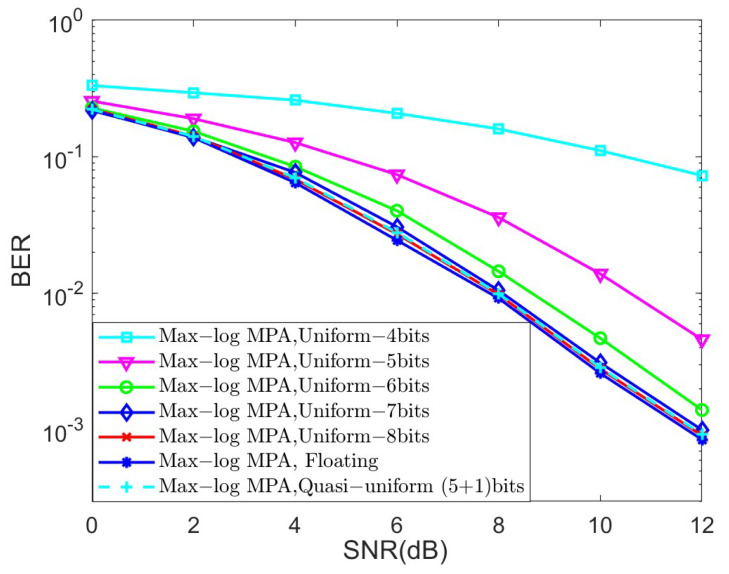
The BER performance of different quantized schemes under the Rayleigh channel for K=5 and the Max-log MPA detector.

We also compared the BER performance of different quantized schemes under the Rayleigh channel for K=4 and K=5 in Figure 12 and Figure 13, respectively, in terms of the Max-log MPA detector. The 4-bit, 5-bit, 6-bit, 7-bit, and 8-bit uniform quantization schemes were considered, where the 8-bit case can achieve nearly the same BER performance as compared to the original Log-MPA. An important observation is that the (5 + 1)-bit quasi-uniform quantization has almost the same BER performance as the 8-bit uniform quantization scheme, which makes the proposed quasi-uniform quantizer more suitable for practical implementation.

## 5. Conclusions

This work proposes the Sub-log MPA detector for the SCMA system by using the latest probabilities at the current iteration to update the messages of the remaining nodes, which reduces both the number of iterations to converge and the cost of hardware design. Next, we describe the conventional uniform quantizer for the SCMA system, whose range of the probabilities is limited. To address this problem, we propose a novel quasi-uniform quantizer, which can effectively extend the dynamic range of the quantizer without modifying the RN and the VN message exchange steps. Simulation results confirmed the robustness of the proposed Sub-log MPA detector and the quasi-uniform quantizer for both the AWGN and Rayleigh channels. Future works may focus on more practical issues such as the quantization of multiple-input multiple-output SCMA and low-density parity-check-coded SCMA systems.

## Figures and Tables

**Table 1:** (4+1)-bit qusi-uniform quantization with Δ=0.5,q=3,μ=2.

Input Range	Quantized Value (Decimal)	Binary Form
[0,0.25)	0	00000
[0.25,0.75)	0.5	00001
[0.75,1.25)	1	00010
[1.25,1.75)	1.5	00011
[1.75,2.25)	2	00100
[2.25,2.75)	2.5	00101
[2.75,3.25)	3	00110
[3.25,3.75)	3.5	00111
[3.75,8)	4	01000
[8,16)	8	01001
[16,32)	16	01010
[32,64)	32	01011
[64,128)	64	01100
[128,256)	128	01101
[256,512)	256	01110
[512,+∞)	512	01111

## Data Availability

Data are contained within the article.
